# Management and outcomes in pregnant patients with monogenic diabetes due to pathogenic variants in *GCK* and *HNF1A* genes

**DOI:** 10.3389/fendo.2025.1687596

**Published:** 2025-12-02

**Authors:** Magdalena Szopa, Karolina Zawadzka, Michał Kania, Przemysław Witek, Katarzyna Cyganek

**Affiliations:** 1 Department of Metabolic Diseases, Jagiellonian University Medical College, Krakow, Poland; 2 University Hospital in Krakow, Krakow, Poland; 3 Doctoral School of Medical and Health Sciences, Jagiellonian University Medical College, Krakow, Poland

**Keywords:** pregnancy, maturity onset diabetes of the young, MODY, monogenic diabetes, MD, GCK, HNF1A

## Abstract

**Background:**

While treatment algorithms for the most common forms of monogenic diabetes (MD) are well established, managing affected pregnancies remains a clinical challenge. This study aimed to evaluate the clinical management and pregnancy outcomes in patients with the two prevalent MD subtypes: GCK and HNF1A.

**Methods:**

We analyzed 36 pregnancies from 27 patients: 18 pregnancies occurred in the context of 14 patients with GCK-hyperglycemia, and 18 pregnancies in 13 patients with HNF1A-MD. Patients’ characteristics, mode of treatment, glycemic control assessed by HbA1c, glycemia and pregnancy outcomes were evaluated.

**Results:**

The mean age of participants was 31.64 ± 3.91 years, similar between groups. Time from the diagnosis of diabetes was longer in subtypes HNF1A-MD (8.00 ± 6.20 vs. 3.46 ± 4.05 years, p=0.046). Preconception BMI and HbA1c were similar between groups. HbA1c during pregnancy was within recommended limits but significantly lower in the HNF1A group during the second trimester (33.2 ± 6.0 vs 38.0 ± 6 mmol/mol, p=0.032). Mean fasting glucose was higher in the GCK-hyperglycemia group in the first trimester (5.6 ± 0.8 vs. 4.9 ± 1.4 mmol/l, p=0.044). Before pregnancy diet therapy predominated in GCK-hyperglycemia (56.0% vs 0%, p<0.001), while insulin therapy was more frequent in HNF1A-MD (67.0% vs. 17.0%, p=0.006). All patients were switched to insulin therapy during pregnancy. Incidences of miscarriages were limited to 2 cases in HNF1A-MD; 1 case of prolonged neonatal hypoglycemia occurred in GCK-hyperglycemia. Maternal and neonatal outcomes were generally favorable.

**Conclusions:**

Pregnancy outcomes in patients with subtypes of monogenic diabetes: GCK-hyperglycemia and HNF1A were comparable and generally favorable. Individualized insulin therapy, regular monitoring and structured outpatient care support safe management even without fetal genotyping, though universal insulin in GCK subtypes diverges from emerging genotype-based practice.

## 1 Introduction

Monogenic diabetes (MD) is a rare, inherited form of diabetes caused by a single pathogenic variant affecting insulin production. Despite its distinct genetic basis, it can be misdiagnosed during pregnancy as gestational diabetes mellitus (GDM), type 1 diabetes (T1D), or type 2 diabetes (T2D). MD accounts for approximately 1–2% of all diabetes cases (1). The most common subtypes of MD are caused by pathogenic variants in *GCK* (GCK-hyperglycemia) or *HNF1A* (HNF1A-MD) genes. Treatment varies depending on the subtype. Patients with HNF1A-MD often respond well to low-dose sulfonylureas, whereas GCK-hyperglycemia is typically mild and may not even meet formal diabetes criteria (2, 3).

Pregnancy presents a unique challenge due to the interplay of maternal glycemia, placental drug transfer, and fetal genotype. Risks of macrosomia, neonatal hypoglycemia, and inappropriate fetal growth may arise if treatment is misaligned with fetal genetic status, particularly in GCK-hyperglycemia (2). Fetal genotyping, either invasive or non-invasive (NIPT/NIPD), is not universally performed (2).

For HNF1A-MD, the safety and timing of sulfonylurea therapy are uncertain, leading to frequent replacement with insulin despite sometimes inferior glycemic control (4, 5). Glibenclamide crosses the placenta and is associated with higher risks of neonatal hypoglycemia and macrosomia in later trimesters (4).

Current clinical practice lacks standardized, evidence based guidelines for MD pregnancies. Misaligned management may lead to adverse outcomes, including miscarriages, preterm delivery, macrosomia, or excessive insulin exposure. Prospective, pathogenic-variant-specific data on maternal and fetal outcomes are limited, particularly when fetal genotyping is unavailable (3).

This study evaluates clinical management and pregnancy outcomes in GCK-hyperglycemia and HNF1A-MD pregnancies focusing on insulin therapy, glycemic control and neonatal outcomes.

## 2 Methods

This retrospective, cohort study was conducted at the Department of Metabolic Diseases, Jagiellonian University Medical College, Poland. All Caucasian pregnant patients with a genetic diagnosis of GCK-hyperglycemia or HNF1A-MD receiving care between February 2010 and January 2020 were included. The inclusion criteria were: 1) genetic diagnosis of GCK-hyperglycemia or HNF1A-MD 2) inpatient medical care initiated during pregnancy 3) complete follow-up until delivery with at least one postpartum visit.

We identified 36 singleton pregnancies (18 GCK-hyperglycemia and 18 HNF1A-MD) from 27 patients (14 patients with GCK-hyperglycemia and 13 patients with HNF1A-MD). Patients selected for genetic testing were characterized by a strong (multigenerational) family history, or additionally, the rapid development of complications, e.g., retinopathy, or in the case of GCK - a characteristic OGTT 75g test result or a persistently elevated fasting glucose level. By the Department’s policy, an auto-antibody panel (anti-GAD, IA-2 and anti-ZnT8) should be ordered to exclude autoimmune etiology of diabetes in a non-typical course hyperglycemia. Patient data were collected while waiting for the opportunity to perform the test.

Baseline characteristics included age, time from the diagnosis of diabetes, family history, weight, body-mass index (BMI), comorbidities, and diabetes-related complications. Retinopathy was assessed via indirect ophthalmoscopy; nephropathy by albumin excretion rate >30 mg/24h or urine albumin-to-creatine ratio >30 mg/g. HbA1c was monitored and structured outpatient care support measured by HPLC (Bio-Rad Variant) and fasting glucose by standard laboratory methods. HbA1c values are presented in mmol/mol (IFCC) and percentage. Laboratory diagnosis of diabetes was made based on WHO criteria (6). Methods used in the molecular diagnosis of MD pathogenic variant in this cohort have been described in detail previously (7).

Routine visits occurred every 2–4 weeks. All patients received structured education on glycemic targets, diet, physical activity, self-monitoring of blood glucose (SMBG), and insulin self-adjustment. Insulin therapy (basal bolus or continuous subcutaneous insulin infusion [CSII]) was individualized. SMBG was recommended ≥ 8 times/day. According to the Diabetes Poland Standards of Care in Diabetes, at the time when the study was performed, the therapeutic targets for all patients were: a) HbA1c <6.0% (42 mmol/l) until 2016, and <6.0% (42 mmol/mmol) in the preconception period and first trimester and <6.5% (48 mmol/mol) in the following trimesters since 2017; b) fasting and pre-prandial SMBG 60–90 mg/dl (3.3-5.0 mmol/l) until 2014 and 70–90 mg/dl (3.9-5.0 mmol/l) since 2015; c) 1-hour postprandial SMBG within <120 mg/dl (6.7 mmol/l) until 2016 and <140 mg/dl (7.9 mmol/l) since 2017 (7). Severe hypoglycemia was defined as an episode of glycemia <70 mg/dl (3.9 mmol/l) requiring medical assistance.

All pregnant patients were covered by specialist obstetric care, including measurement of the fetus’s size, at the University Hospital in Krakow. Macrosomia was defined as a birth weight greater than 4000 g and low birth weight was defined as a birth weight of less than 2500 g. Due to not complete data on the growth of newborns, LGA could not be reported. Obstetric outcomes included were miscarriages, mode of delivery, preterm birth, and cesarean section.

After delivery, patients were encouraged to schedule a follow-up in the clinic 1 month postpartum and then approximately every 3 months subsequently. During these visits, glycemic control and weight were assessed. Patients were again instructed on how to perform SMBG and information was provided by a physician on postdelivery glycemic goals and diet. Additional sessions with a dietitian were scheduled if necessary.

Normally distributed data were analyzed with t-tests, non-normal data with Mann-Whitney test, and categorical data with Fisher’s exact test. Repeated pregnancies in the same patient were accounted for using mixed-effects models. Significance was set at p<0.05. Analyses were performed in StatSoft Statistica v.13 (StatSoft Inc., Tulsa, OK, USA).

This study was based entirely on a retrospective analysis of patients’ medical records, and ethics approval was therefore waived. The analysis did not affect any diagnostic procedures or treatment methods. As such, this type of research is exempt from obtaining informed consent.

## 3 Results

36 singleton pregnancies (18 GCK-hyperglycemia and 18 HNF1A-MD) from 27 patients (14 patients with GCK-hyperglycemia and patients with HNF1A-MD, **Table 1**).

**Table 1:** Pathogenic/likely pathogenic variants with cDNA and protein nomenclature in study participants.

GCK-hyperglycemia			
Patient number	Number of pregnancies	cDNA pathogenic/likely pathogenic variants (heterozygous)	Amino acid change
1	1	c.[952G>A];[=]	p.(Gly318Arg);p(=)
2	1	c.[952G>A];[=]	p.(Gly318Arg);p(=)
3	2	c.[952G>A];[=]; c.[645C>T];[=]	p.(Gly318Arg);p(=)
4	1	c.[773G>A];[=]	p.(Gly258Asp);p(=)
5	1	c.[952G>A];[=]	p.(Gly318Arg);p(=)
6	1	c.[944T>A];[=]	p.(Leu315His);p(=)
7	2	c.[1148C>T];[=]	p.(Ser383Leu);p(=)
8	1	c.[952G>A];[=]	p.(Gly318Arg);p(=)
9*	2	c.[170T>C];[=]/c.[182T>G];[=]	p.(Met57Thr);p(=)/p(Val61Gly);p(=)
10	2	c.[491T>C];[=]; c.[213C>T];[=]	p.(Leu164Pro);p(=)
11	1	c.[370G>A];[=]	p.(Asp124Asn);p(=)
12	1	c.[952G>A];[=]	p.(Gly318Arg);p(=)
13	1	c.[952G>A];[=]	p.(Gly318Arg);p(=)
14	1	c.[490C>G];[=]	p.(Leu164Val);p(=)
HNF1A-MD			
15	1	c.[1137del];[=]	p.(Val380Serfs*4);p(=)
16	2	c.[770A>C];[=]	p.(Asn257Thr);p(=)
17	1	c.[872dup];[=]	p.(Gly292Argfs*25);p(=)
18	1	c.[745T>C];[=]	p.(Ser249Pro);p(=)
19	1	c.814C>T;[=]	p.(Arg272Cys);p(=)
20	2	c.[1004del];[=]	p.(Ser335*);p(=)
21	1	c.[182T>G];[=]	p(Gly292Argfs*);p(=)/p(Val61Gly);(=)
22	1	c.[1502-6G>A];[=]	
23	1	c.[197dup];[=]	p.(Thr67Aspfs*29);p(=)
24	2	c.[779C>T];[=]	p.(Thr260Met);p(=)
25	1	c.[824A>T];[=]	p.(Glu275Val);p(=)
26	2	c.[811C>T];[=]	p.(Arg271Trp);p(=)
27	2	Unidentified&	

* In participant number 9 from GCK-group two pathogenic variants were identified: c.[170T>C];[=] for *GCK* and c.[182T>G];[=] for *HNF1B.*

*&* In participant number 27 genetic testing in progress.

MD, Monogenic Diabetes.

The baseline characteristics of study participants are presented in **Table 2**. The mean age was 31.64 ± 3.91 years. BMI and HbA1c were similar between groups. HNF1A-MD patients had longer time from the diagnosis of diabetes (8.0 vs 3.5 years, p=0.046). Retinopathy present in two HNF1A-MD patients; none of the GCK-hyperglycemia patients had diabetic complications.

**Table 2:** Baseline clinical characteristics of pregnancies in the study participants with GCK-hyperglycemia and HNF1A-MD.

Outcomes	GCK-hyperglycemia	HNF1A-MD	p
Number of pregnancies analyzed	18	18	–
Time from MD diagnosis (years)	3.46 ± 4.05	8.00 ± 6.20	0.046
Pregnancy planning, n (%)	7 (38.00)	9 (50.00)	0.740
Age at time of MD diagnosis (years)	30.00 ± 4.98	29.40 ± 4.12	0.760
Preconception body weight (kg)	60.76 ± 8.62	64.67 ± 14.83	0.360
Preconception BMI (kg/m^2^)	22.11 ± 2.95	24.63 ± 6.35	0.558
Preconception HbA1c (mmol/mol, %)	45 ± 4 6.29 ± 0.30	48 ± 23 6.55 ± 2.10	0.265
Treatment before pregnancy: insulin, n (%)	3 (16.67)	12 (66.67)	0.006
Treatment before pregnancy: sulphonylureas, n (%)	1 (5.56)	3 (16.67)	0.603
Treatment before pregnancy: diet alone, n (%)	10 (55.56)	0 (0)	<0.001
Patients without diabetes treatment before pregnancy (n)	4 (22.2)	3 (16.67)	1.000
Retinopathy, n (%)	0 (0)	2 (11.11)	0.230
Neuropathy, n (%)	0 (0)	1 (5.56)	0.310
Nephropathy, n (%)	0 (0)	0 (0)	–

MD, Monogenic Diabetes; BMI, body mass index.

The treatment of GCK-hyperglycemia patients before pregnancy significantly differed from that of HNF1A-MD patients. Diet alone was more common in GCK-hyperglycemia (55.6% vs 0%, p<0.001). Insulin therapy was more frequent in HNF1A-MD (67% vs 17%, p=0.006). Sulfonylureas were used in 17% of HNF1A-MD patients and in 6% of GCK-hyperglycemia patients; all of whom were switched to insulin during pregnancy. Insulin treatment in pregnancy was introduced earlier in the HNF1A-MD group than in the GCK-hyperglycemia group, however, this difference was not statistically significant (7.50 vs. 13.00 week, p=0.640). During the first and second trimester, apart from one woman from the HNF1A-MD group treated with bolus insulin only, all patients were treated with basal-bolus insulin therapy. During the third trimester, apart from one woman from the GCK-hyperglycemia group treated with basal insulin only, all patients were treated with basal-bolus insulin therapy. GCK-hyperglycemia patients received a higher total daily dose of insulin during the first trimester than HNF1A-MD patients (0.54 ± 0.14 vs. 0.41 ± 0.17 units/kg, p=0.041). Moreover, GCK-hyperglycemia patients received numerically higher total daily dose of insulin in every trimester compared to HNF1A-MD patients, but the differences were not statistically significant (**Table 3**). The daily dosage of basal insulin was higher in the GCK-hyperglycemia group in the first and second, but not third trimester (**Table 3**). There were 4 (11%) pregnant patients treated with CSII during pregnancy: 2 with GCK-hyperglycemia and 2 with HNF1A-MD. Three patients were switched to CSII during pregnancy and only 1 woman with HNF1A-MD had been receiving CSII therapy during the pregnancy planning time preceding pregnancy.

**Table 3:** Clinical maternal outcomes in the GCK-hyperglycemia and HNF1A-MD groups.

Outcome	GCK-hyperglycemia	HNF1A-MD	p
Age at time of pregnancy (years)	30.89 ± 4.74	32.61 ± 2.75	0.190
Week of gestation at the time of first visit (weeks)	9.00 (8.00-14.00)	7.00 (6.00-13.00)	0.280
HbA1c - 1^st^ trimester (mmol/mol, %)	42.7 ± 3.10 6.06 ± 0.29	41.0 ± 12.00 5.90 ± 1.10	0.180
HbA1c - 2^nd^ trimester (mmol/mol, %)	38.5 ± 5.20 5.65 ± 0.48	32.7 ± 5.90 5.13 ± 0.54	0.032
HbA1c - 3^rd^ trimester (mmol/mol, %)	36.3 ± 6.60 5.46 ± 0.60	34.2 ± 4.60 5.26 ± 0.42	0.390
Mean SMBG glucose - 1^st^ trimester (mg/dl)	109.55 ± 12.88	104.23 ± 15.72	0.370
Mean SMBG glucose - 2^nd^ trimester (mg/dl)	113.61 ± 10.99	107.55 ± 13.94	0.260
Mean SMBG glucose - 3^rd^ trimester (mg/dl)	113.35 ± 11.26	105.93 ± 13.69	0.140
Mean fasting glucose - 1^st^ trimester (mg/dl)	100.55 ± 15.28	87.78 ± 24.58	0.044
Mean fasting glucose - 2^nd^ trimester (mg/dl)	98.69 ± 15.49	88.57 ± 8.48	0.130
Mean fasting glucose - 3^rd^ trimester (mg/dl)	91.08 ± 17.53	102.83 ± 31.24	0.310
Point of insulin introduction during pregnancy (weeks of gestation)	13.00 (8.5-23.5)	7.50 (5.00-32.00)	0.640
HbA1c at the time of first visit (mmol/mol, %)	43.2 ± 3.8 6.10 ± 0.35	40.9 ± 11.7 5.87 ± 1.07	0.120
Total daily insulin dose - 1^st^ trimester (units)	34.61 ± 9.49	29.43 ± 15.92	0.330
Total daily insulin dose - 1^st^ trimester (units/kg)	0.54 ± 0.14	0.41 ± 0.17	0.041
Basal insulin 1^st^ trimester (%)	40.43 ± 13.75	27.48 ± 13.61	0.027
Total daily insulin dose - 2^nd^ trimester (units)	49.85 ± 13.4	48.56 ± 36.42	0.156
Total daily insulin dose - 2^nd^ trimester (units/kg)	0.73 ± 0.21	0.63 ± 0.29	0.289
Basal insulin 2^nd^ trimester (%)	33.07 ± 11.54	20.69 ± 12.00	0.017
Total daily insulin dose - 3^rd^ trimester (units)	72.31 ± 35	67.16 ± 45.85	0.380
Total daily insulin dose - 3^rd^ trimester (units/kg)	0.97 ± 0.43	0.82 ± 0.44	0.385
Basal insulin 3^rd^ trimester (%)	38.64 ± 20.09	24.95 ± 13.84	0.094
Weight gain during pregnancy (kg)	16.59 ± 5.23	11.62 ± 7.69	0.087
BMI at the end of pregnancy (kg/m^2^)	27.62 ± 3.12	29.4 ± 5.27	0.330

MD, Monogenic Diabetes; SMBG, self monitoring blood glucose; BMI, body mass index.

**Figure 1:**
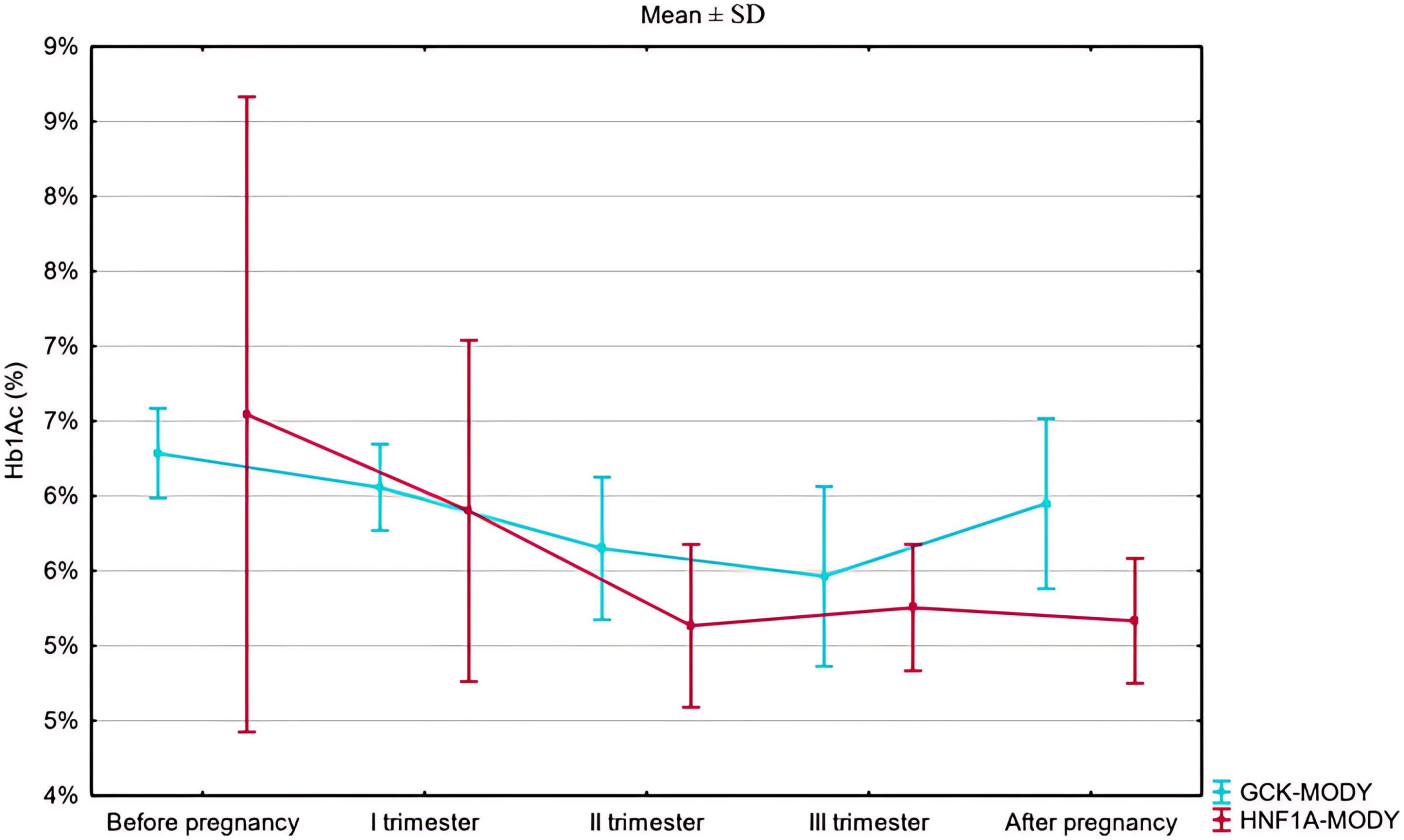
Temporal trends in HbA1c before, during, and after pregnancy.

HbA1c was numerically higher in the HNF1A-MD group than in the GCK-hyperglycemia group prior to gestation (48 ± 23 mmol/mol [6.55 ± 2.10%) vs. 45 ± 4 mmol/mol [6.29 ± 0.30%], p=0.264, **Figure 1**, **Table 2**). HbA1c during pregnancy was within recommended limits in both groups but significantly lower in HNF1A-MD patients during the second trimester (32.7 ± 5.9 mmol/mol [5.13 ± 0.54%] vs. 38.5 ± 5.2 mmol/mol [5.65 ± 0.48%], p=0.03, **Table 3**).

**Figure 2:**
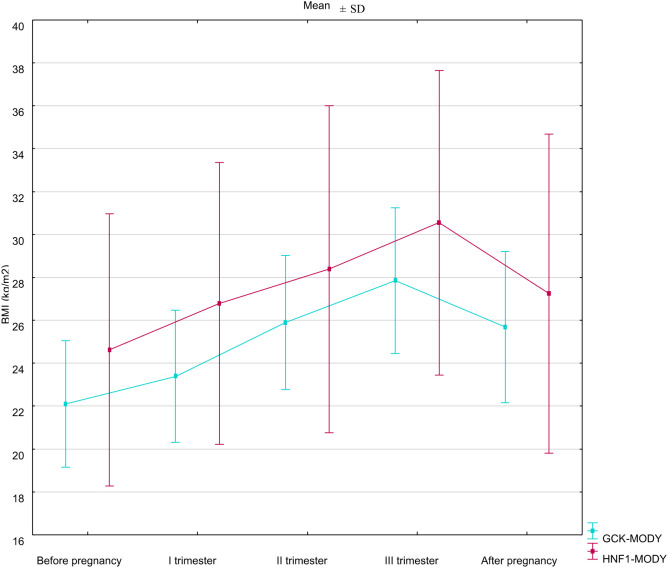
Temporal trends in BMI before, during, and after pregnancy.

There were no statistically significant differences between GCK-hyperglycemia and HNF1A-MD groups regarding mean SMBG in any trimester of pregnancy (**Table 3**). Mean fasting glucose was significantly higher in GCK-hyperglycemia in the first trimester when compared to the HNF1A-MD group. We did not observe differences between the two groups in fasting glucose levels in the second and third trimesters (**Table 3**, **Figure 2**).

There were no incidences of severe hypoglycemia in GCK-hyperglycemia and HNF1A-MD pregnancies. There were no differences in the duration of pregnancy and mean newborn birth weights (3412.5 vs. 3307g, p=0.59). There were two macrosomic neonates delivered in the HNF1A-MD group and three in the GCK-hyperglycemia group, with no low birth weight deliveries as shown in more detail in **Table 4**. The rates of caesarean deliveries in subtypes of GCK-hyperglycemia and in HNF1A-MD were similarly high. There was one preterm delivery noted in each of the HNF1A-MD and GCK-hyperglycemia groups. Two miscarriages occurred among HNF1A-MD patients. Three congenital disorders (ventricular septal defect, dilated pelvicalyceal system and hydrocephalus) in the HNF1A-MD group and one complication (prolonged neonatal hypoglycemia) in the GCK-hyperglycemia group were noted.

**Table 4:** Clinical perinatal outcomes in subtypes the GCK-hyperglycemia and HNF1A-MD groups.

Outcome	GCK-hyperglycemia	HNF1A-MD	p
Gestational age at the time of delivery (weeks)	38.50 (38-39)	39.00 (38-39)	0.610
Birth weight in at-term pregnancies (g)	3412.50 ± 568.38	3307.00 ± 450.57	0.590
Caesarean delivery, n (%)	10 (83.30)	13 (81.25)	0.890
Preterm deliveries, n (%)	1 (7.69)	1 (6.25)	
Miscarriages, n (%)	0 (0.00)	2 (11.10)	
Infant death, n (%)	0 (0.00)	0 (0.00)	
Malformations, n (%)	1 (8.33)	3 (18.75)	
Macrosomia, n (%)	3 (20.00)	2 (11.1.00)	

MD, Monogenic Diabetes.

Information from the post-delivery follow-up period was only available for 6 patients with GCK-hyperglycemia and 7 patients with HNF1A-MD. In 3 patients with GCK-hyperglycemia and in 1 with HNF1A-MD, insulin therapy was discontinued at the follow-up visit.

## 4 Discussion

This study provides a detailed comparison of pregnancies affected by GCK-hyperglycemia and HNF1A-MD. This study contributes to the limited literature by providing a detailed, direct comparison of clinical management, insulin requirements, and maternal and neonatal outcomes in pregnancies affected by these two most common MD subtypes.

Although pathogenic variants in both *GCK* and *HNF1A* genes are well-recognized causes of MD (8), only one prior retrospective study—by Bacon et al. (9)—has compared pregnancy outcomes between them. Our results confirm several of their key findings. As in Bacon’s cohort, HNF1A-MD pregnancies in our study were generally easier to manage in terms of achieving glycemic targets. In contrast, patients with GCK-hyperglycemia required significantly higher insulin doses — particularly in the second trimester — yet achieved poorer glycemic control. This reflects a known pathophysiological difference: relative insulin resistance and a higher glycemic set point in GCK-hyperglycemia patients. According to previous reports, in GCK-hyperglycemia pregnant patients supraphysiological insulin doses (>1 unit/kg/day) are usually required to overcome maternal counterregulatory mechanisms (2).

However, our study also extends prior findings. Unlike in Bacon et al. and other reports such as Spyer et al. (10), where many GCK-hyperglycemia patients were managed conservatively with diet alone, all patients in our cohort — including those with GCK-hyperglycemia — were treated with insulin throughout pregnancy. This reflects both institutional practice and the challenges posed by an unknown fetal genotype. When the fetus inherits the *GCK* pathogenic variant, maternal hyperglycemia typically does not lead to fetal overgrowth. However, if the fetus does not carry the pathogenic variant, untreated maternal hyperglycemia may contribute to macrosomia (2). In our center, the decision to initiate insulin was made empirically, given the absence of genotypic data, and therapy was titrated to meet fasting and postprandial glucose targets. To mitigate the risk of both excess maternal weight gain and small for gestational age birthweight in ca. 50% children that would inherit *GCK* pathogenic variant, a close ultrasound monitoring of the fetus’s size was performed.

Notably, despite more challenging glycemic control in the GCK-hyperglycemia group, neonatal outcomes were not worse. In the HNF1A-MD group, there were 2 miscarriages, while in GCK-hyperglycemia group - one case of prolonged neonatal hypoglycemia. Miscarriages in the HNF1A-MD group were are most likely related to higher maternal glucose levels at the beginning of pregnancy, while neonatal hypoglycemia is more likely related to maternal hyperinsulinism in patients with GCK-hyperglycemia. The incidence of macrosomia — 20% GCK-hyperglycemia and 11.1% in HNF1A-MD—was lower than previously reported in both subtypes (9, 10). Mean birth weights and gestational durations were comparable. These findings suggest that regular monitoring and appropriate insulin adjustments can mitigate risks even in the absence of fetal genotyping.

Insulin regimens in both groups were highly individualized. While no unified protocol was used, all patients were monitored closely with dose titration based on SMBG profiles. This mirrors real-world practice, especially in settings without standardized MD-specific guidelines. In GCK-hyperglycemia, the need for higher total daily insulin doses — despite suboptimal HbA1c in the second trimester — highlights physiological resistance to insulin therapy due to homeostasis and supports the use of tailored approaches.

In contrast, patients with HNF1A-MD responded well to insulin. Although sulfonylureas such as glibenclamide are typically used outside pregnancy and considered relatively safe during early gestation, concerns about placental transfer and fetal hypoglycemia in later trimesters led us to switch all patients to insulin (4). This approach was consistent with individualized care and local practice patterns. While some evidence suggests sulfonylureas may offer better glycemic control in early pregnancy, we prioritized insulin later on to avoid fetal overgrowth.

Our findings also emphasize the central role of patient education and multidisciplinary outpatient care. Pre-pregnancy counselling, shared decision-making regarding treatment plans, and lifestyle modification were key elements in achieving favorable outcomes. Given that MD remains underrecognized and often misclassified, integrating MD-specific content into diabetology and obstetrics training is critical—particularly in settings where genetic testing is limited.

Furthermore, our study underscores the clinical dilemma posed by the lack of fetal genotyping. Invasive procedures carry risks and are rarely performed. However, emerging non-invasive prenatal testing (NIPT) strategies for monogenic diabetes may soon allow for safer and earlier identification of fetal genotype. Until such methods are widely available, serial fetal ultrasound from 26 weeks remains the standard recommendation to detect early signs of macrosomia (2). Still, due to the trend of increasing pre-pregnancy overweight and obesity or excessive gestational weight gain, all of which accelerate the increase of fetal abdominal circumference, the ultrasound may not be fully reliable (2).

This study is limited by its retrospective design and relatively small sample size, inherent to the rarity of MD in pregnancy. The diagnosis of monogenic diabetes was known in approximately two-thirds of the women – for GCK-hyperglycemia - 9 women, and HNF1A-MD - 9 women. In the remaining cases, the lack of information regarding diabetes type and the absence of treatment before pregnancy were most commonly related to unawareness of the underlying condition. The absence of fetal genotype data precludes definitive conclusions regarding genotype-phenotype correlations in offspring. Additionally, changes in clinical practice over time may have influenced therapeutic decisions (11). The data analysis in our work covers the years from 2010 to 2018. Initially, we relied only on data regarding the glycemia target during pregnancy, which before 2014 was: fasting glycaemia: 3,99 – 4,99 mmol/l (70–90 mg/dl) and 6,7 mmol/l (120 mg/dl) 1 hour after a meal. Indeed, initially, at our center, we tried to achieve normoglycemia during pregnancy in accordance with the then-current recommendations of the Diabetes Poland, which differed from global recommendations and were more stringent. After 2014, the recommendations in Poland also changed and currently do not differ from global standards, including those regarding the treatment of patients with suspected GCK-hyperglycemia and avoiding overtreatment and overinsulinization Moreover, in accordance with the consensus of Diabetes Poland, glibenclamide should not be used as first-line therapy in pregnancy, as it crosses the placenta and is associated with neonatal hypoglycemia and macrosomia. It was highlighted that in our center as all patients on sulfonylureas were switched to insulin to minimize the risk. Another limitation is the small number of patients and pregnancies (27 and 36, respectively); therefore, the analysis of rare events (congenital malformations, neonatal hypoglycemia, preterm birth) has limited statistical power to assess the effect of the MD phenotype on peri-obstetric complications. Nonetheless, our results reflect real-world experience in a specialized center and demonstrate the feasibility of safe, individualized care in MD pregnancies.

In conclusion, maternal and neonatal outcomes in pregnancies affected by GCK-hyperglycemia and HNF1A-MD were largely favorable and comparable between groups. Our findings are consistent with previous reports and further highlight key clinical distinctions between the subtypes of MD, particularly in terms of insulin requirements and glycemic response. Individualized insulin therapy, regular monitoring, and structured outpatient care — along with future access to non-invasive fetal genotyping—may enhance precision in managing MD pregnancies. Greater clinical awareness and adapted care pathways are essential, especially in regions with limited access to genetic testing. Future integration of non-invasive fetal genotyping may further improve management precision.

## Data Availability

The raw data supporting the conclusions of this article will be made available by the authors, without undue reservation.
